# Population-Specific Mutational Spectrum of Autosomal Recessive Nonsyndromic Hearing Loss in Croatian Roma: Implications for Clinical Genetics

**DOI:** 10.3390/genes17040468

**Published:** 2026-04-16

**Authors:** Iva Kutija Fučkar, Matea Zajc Petranović, Irena Martinović Klarić, Marijana Peričić Salihović, Lovorka Barać Lauc

**Affiliations:** 1 Institute of Immunology, 10000 Zagreb, Croatia; ifuckar@imz.hr; 2 Institute for Anthropological Research, 10000 Zagreb, Croatia; matea@inantro.hr (M.Z.P.); mpericic@inantro.hr (M.P.S.); 3 Institute for Migration Research, 10000 Zagreb, Croatia; irena.martinovic.klaric@imin.hr

**Keywords:** hereditary hearing loss, GJB2, Roma population, c.71G>A, c.457G>A, c.380G>A, c.109G>A, c.269T>C

## Abstract

Background/Objectives: Hearing impairment is a highly prevalent sensory disorder resulting from a variety of causes. A high proportion of autosomal recessive non-syndromic hearing impairment is linked to the *GJB2* (OMIM 121011) gene which encodes for a gap junction protein, connexin-26. Alterations of genes that encode for connexins can lead to changes in cell ion content and cause hearing impairment. Methods: *GJB2* gene polymorphisms (c.71G>A, p.Trp24*rs104894396; c.457G>A, p.Val153Ile, rs111033186; c.380G>A, p.Arg127His, rs111033196; c.109G>A, p.Val37Ile, rs72474224; and c.269T>C, p.Leu90Pro, rs80338945) were analyzed in the Roma population of Croatia. Loci were genotyped using the KASP method. Results: Altered alleles were detected on the loci c.71G>A, c.457G>A and c.380G>A and statistically significant differences in allele frequencies were noticed. Furthermore, in comparison to worldwide populations, the Roma population also shows statistically significant difference in allele frequency of these loci. Conclusions: This study reveals marked genetic differentiation among Croatian Roma particularly with respect to the c.71G>A variant. Characterizing such population-specific mutational heterogeneity is crucial for the accurate prevention, diagnosis, and clinical management of autosomal recessive nonsyndromic hearing loss.

## 1. Introduction

### 1.1. Hereditary Deafness

Sensorineural hearing loss (SNHL) constitutes a significant public health concern worldwide, substantially impacting the quality of life of affected individuals, especially when it develops early in life. According to the World Health Organization (WHO) data from 2025, more than 5% of the world’s population has hearing loss, which amounts to approximately 430 million people, including 34 million children. Estimates from 2023 indicate that the number of people with hearing impairment could exceed 700 million by 2050 [1]. Disabling hearing loss is defined as a hearing loss greater than 35 decibels in the better ear. In children, hearing loss is the most common sensory disorder, with a prevalence of up to 3 per 1000 newborns, and in more than half of the cases, the cause is genetic [2,3].

Hereditary deafness can manifest as syndromic or nonsyndromic, with the latter accounting for approximately 70% of genetically caused hearing loss cases [4]. Among more than 90 genes involved in the etiology of nonsyndromic deafness, *GJB2* (gap junction beta-2 protein gene), which encodes the protein connexin-26, plays a key role in potassium ion homeostasis within the cochlea, essential for sound transduction in the inner ear [5]. Mutations in the *GJB2* gene are one of the most common causes of autosomal recessive nonsyndromic deafness (DFNB1, MIM 220290).

### 1.2. Population-Specific Mutations in the GJB2 Gene

The *GJB2* gene consists of two exons, similar to most genes encoding connexins. Exon 1 is non-coding, so the entire coding region is located within exon 2, which is separated from the 5′-untranslated region by an intron of variable length. Exon 2 is 681 base pairs long and encodes a protein composed of 226 amino acids. More than 300 variants of the *GJB2* gene have been described as underlying causes of autosomal recessive nonsyndromic hearing loss (ARNSHL) [6]. Notably, the prevalence of GJB2 polymorphisms varies significantly across populations, and population history has been proposed to play a considerable role in shaping the distribution of GJB2 variants and the incidence of associated hearing loss [7]. Recurrent *GJB2* mutations often show ethno-geographic specificity, with certain pathogenic alleles being highly frequent in particular regions or populations. These patterns highlight how demographic history and genetic drift have shaped the present-day distribution of GJB2 mutations globally [8]. Several population-specific founder mutations have been identified worldwide, such as c.35delG in Europeans, c.167delT in Ashkenazi Jews, c.235delC in East Asian populations, and c.71G>A in South Asian populations. In addition, in Roma populations, a distinctive *GJB2* variant spectrum has been reported; for example, the c.71G>A is highly prevalent among Romani groups in Spain and other European regions, reflecting an ancestral founder allele that contributes substantially to ARNSHL in these isolates [9,10]. Population-specific allele frequencies and genotype–phenotype correlations of *GJB2* variants have also been highlighted in recent reviews of global *GJB2*-associated hearing loss, underscoring the importance of ethnicity in the prevalence and clinical impact of hearing loss.

### 1.3. GJB2 Gene Mutations in Roma Populations

The Roma population is a transnational minority living in numerous countries worldwide, whose gene pool is strongly shaped by specific genetic history, notably the founder effect, reproductive isolation and limited exogamy in the more distant past [11]. As a result of diverse social and economic pressures in European countries, the Roma have slowly divided into many geographically scattered groups with unique social, linguistic and genetic characteristics.

Genetic studies of Roma populations have identified 90 autosomal recessive disorders and 111 pathogenic variants attributable to population-specific private mutations [12]. Regarding hereditary deafness in Roma subpopulations, genetic studies are largely restricted to analyses of the *GJB2* gene. Studies in Spanish and Slovak Roma cohorts indicate that *GJB2* mutations account for up to 50% of autosomal recessive nonsyndromic hearing loss (ARNSHL) cases in these populations, underscoring the major contribution of this locus. This high prevalence is primarily driven by the c.71G>A variant, which alone has been reported to explain 39.5% of ARNSHL cases in Spanish Roma and 23.2% in Slovak Roma individuals [9,13]. Its high prevalence in Indian and Pakistani populations supports the hypothesis of a founder effect introduced into Europe during the historical migration of Roma from the Indian subcontinent [13]. The same variant has also been detected at a high frequency among Czech patients with hereditary deafness of Roma ancestry (9.7%) [14]. Carrier frequencies of c.71G>A in Roma control populations have been estimated at 4–5% across major linguistic and migratory groups, including Balkan, Vlax, and Western European Roma populations [10]. However, population-specific differences have been observed, as evidenced by the substantially lower carrier frequency reported in Hungarian Roma controls (0.8%) [15]. In addition to c.71G>A), other *GJB2* variants, such as c.380G>A and c.35delG, have been identified in Slovak Roma patients, accounting for 19.4% and 8.3% of ARNSHL cases, respectively, while c.35delG has been reported in 8.5% of ARNSHL cases among Spanish Roma individuals [9,13].

### 1.4. Roma in Croatia

The presence of Roma in present-day Croatia was first recorded in 1362 in Dubrovnik merchant documents. The Croatian Roma population includes Balkan Roma and descendants of Vlax Roma, who speak an archaic Romanian dialect and are referred to as Vlax Roma or Bayash. While traditionally nomadic, the Bayash population in northwestern Croatia is largely sedentary and autochthonous. They exhibit high endogamy, marrying primarily within the group, and maintain a tradition of large families.

**Figure 1:**
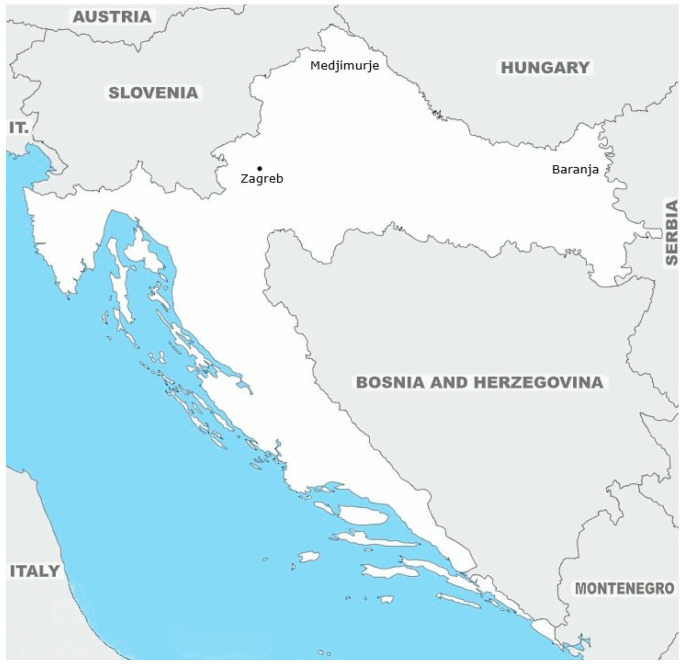
Sampling locations.

Croatian Roma are socio-culturally and linguistically distinct: Vlax Roma speak the ljimb’dbayash language, and Balkan Roma speak dialects of Romani Chib. Within the Bayash, subdialectal differences divide them into Mućani Roma, inhabiting Baranja, and Erdelyi Roma, residing in Medjimurje (Figure 1). In designing our sampling strategy, we aimed to encompass the major Roma groups residing in Croatia in order to capture the existing socio-cultural and linguistic diversity within the Croatian Roma population. Despite this internal differentiation, the Bayash form a cohesive group distinct from other Roma populations [11].

These socio-cultural distinctions and historical isolation have contributed to local founder effects and population-specific genetic structure, e.g., [16,17].

Despite the significant importance of *GJB2* polymorphisms in clinical hearing genetics, studies investigating their frequency in Croatian Roma groups have not been conducted so far. The lack of such data limits precise genetic counseling and targeted diagnostic approaches, which may have consequences in the context of early detection and rehabilitation of hearing impairment. Therefore, the objective of this study is to assess the carrier frequency of five *GJB2* polymorphisms in the Croatian Roma population and to examine their distribution among the three major Croatian Roma groups characterized by distinct socio-cultural and genetic backgrounds.

## 2. Materials and Methods

### 2.1. Sample

We analyzed 434 DNA samples, all collected during field studies of the ongoing multidisciplinary anthropological, molecular–genetic and epidemiological investigations of the Roma populations in Croatia. Samples belong to members of the three socio-culturally different Roma subpopulations: Vlax Roma, who are divided into two subpopulations according to the geographical regions of Croatia they inhabit: Baranja and Medjimurje; and Balkan Roma from the city of Zagreb (Figure 1). The sample sizes of all three Roma population groups were evenly distributed, comprising 132 Vlax Roma from Baranja, 131 Vlax Roma from Medjimurje, and 177 Balkan Roma from the City of Zagreb.

All Roma individuals participated in the study voluntarily. Prior to enrollment, and with the assistance of the Roma community volunteers, participants were thoroughly informed about the objectives, methodology, and expected outcomes of the study in a culturally sensitive manner. Informed consent was obtained from all participants. The study protocol was reviewed and approved by the Scientific Council and the Ethics Committee of the Institute for Anthropological Research, Zagreb, Croatia.

### 2.2. Genetic Analyses

DNA was extracted from peripheral blood using the salting-out method [18]. The genotyping of 5 SNPs in the *GJB2* gene was carried out using the Kompetitive Allele-Specific PCR method (KASP) in a commercial facility. The KASP genotyping assay is a form of the competitive allele-specific PCR combined with a homogeneous fluorescent SNP genotyping system, which determines the alleles at a specific locus within genomic DNA [17].

### 2.3. Statistical Analyses

Allele and genotype frequencies were calculated by direct counting. The Hardy–Weinberg equilibrium (HWE) was assessed using the Arlequin 3.5 software [19]. Genotype and allele frequency differences between the three Roma groups were tested using the Chi-square test and the Bonferroni correction for multiple testing was applied.

For comparative analyses with populations from other geographic regions, reference allele frequency data were obtained from the gnomAD database v4.1.0 (https://gnomad.broadinstitute.org/) (accessed on 12 August 2025). Although the gnomAD population labels are imperfect, do not fully represent all ancestries, and allele frequencies may not be reliable for under-represented or highly structured populations, it remains the most comprehensive database currently available. Therefore, it was used as a proxy for worldwide population variation.

## 3. Results

First, genotype and allele frequencies of three polymorphic variants in the *GJB2* gene (c.71G>A, p.Trp24*, rs104894396; c.457G>A, p.Val153Ile, rs111033186; c.380G>A, p.Arg127His, rs111033196) were analyzed in three Croatian Roma groups (Balkan, Medjimurje, and Baranja) and in the total sample (Table 1). For c.457G>A (rs111033186), the G:G genotype was predominant in all groups (≥97%). The heterozygous G:A genotype was observed only in the Balkan group (1.12%), while the A:A genotype was not detected. The overall frequency of the A allele was 0.56%. No statistically significant differences in genotype distributions were observed among the groups (χ^2^ = 2.93, *p* = 0.231). For c.380G>A (rs111033196), the C:C genotype was the most frequent in the total sample (68.97%). The C:T genotype ranged from 16.76% in the Balkan group to 40.94% in the Medjimurje group, while the T:T genotype was present at low frequencies in all groups. The overall frequency of the T allele was 17.36%. Genotype distributions differed significantly in the overall comparison of the groups (χ^2^ = 34.66, *p* = 5.47 × 10^−2^), but not after Bonferroni correction. For c.71G>A (rs104893396), the G:G genotype predominated in all groups. The G:A genotype was observed in the Balkan and Baranja groups but was not detected in the Medjimurje group, and the A:A genotype was not observed in any group. The overall frequency of the A allele was 2.3%. Statistically significant differences in genotype distributions were observed in the overall comparison of the groups, even after Bonferroni correction (χ^2^ = 10.55, *p* = 0.0051).

Two out of five initially investigated SNPs in the *GJB2* gene were found to be monomorphic (c.109G>A, p.Val37Ile, rs72474224 and c.269T>C, p.Leu90Pro, rs80338945). All three polymorphic loci were in the Hardy–Weinberg equilibrium.

**Figure 2:**
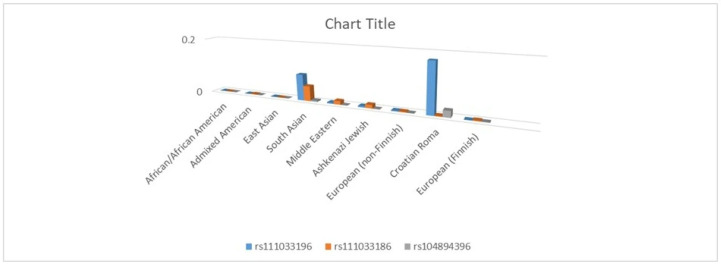
Frequency of altered alleles in rs1110033196, rs111033186 and rs104894396.

In order to compare the allele distribution of three polymorphic SNP with other populations, we searched the gnomAD database for worldwide frequencies of the investigated SNPs. Figure 2 shows the distribution of allele frequencies for three *GJB2* polymorphic variants—c.380G>A (rs111033196), c.457G>A (rs111033186), and c.71G>A (rs104893396)—across worldwide reference populations from the gnomAD database and the Croatian Roma population. The c.380G>A variant (rs111033196) exhibited the highest allele frequency in the Croatian Roma population compared with other analyzed populations, while lower frequencies were observed in South Asian and European populations. The c.457G>A variant (rs111033186) was detected at low frequencies, primarily in South Asian populations, and was rare or absent in most other populations. The c.71G>A variant (rs104893396) showed low allele frequencies across all populations, with slightly higher values observed in the Croatian Roma population compared with other European groups. African/African American, Admixed American, East Asian, Middle Eastern, Ashkenazi Jewish, and European (Finnish and non-Finnish) populations exhibited low or near-zero frequencies for all three analyzed variants.

Two monomorphic SNPs in the Roma population have frequencies below one percent in all worldwide populations except c.109G>A (rs72474224) in east Asians, where altered allele has a frequency of almost 5%. In fact, the altered variant shows a markedly higher frequency in the East Asian population compared with all other populations, in which both variants exhibit very low frequencies.

**Figure 3:**
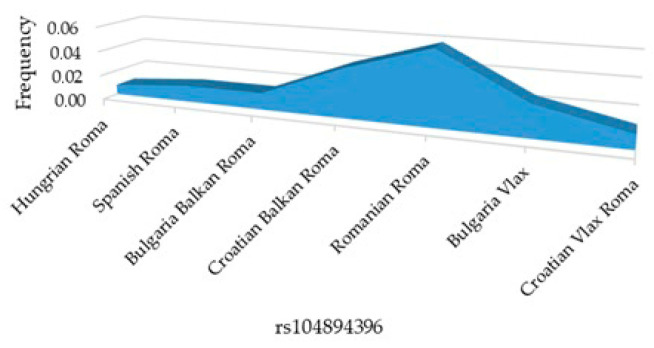
Frequency of altered allele in rs104894396 in European Roma populations.

Among polymorphic SNPs, c.71G>A (rs104894396) has been the most studied in Roma populations. The area plot in Figure 3 shows that the c.71G>A (rs104894396) allele frequency is modest in the majority of analyzed groups. However, c.71G>A shows the highest frequency in Romanian Roma, followed by intermediate frequencies in Bulgaria Vlax and Croatian Vlax Roma.

Our analyses did not show statistically significant LD among the studied loci (rs104894396, rs111033186, rs111033196) in any of the Roma groups from Croatia.

## 4. Discussion

Genetic and biomedical research conducted over the past two decades has provided important insights into the population structure and health status of the Croatian Roma. Early genetic studies based on uniparental markers reveal significant differences in haplogroup distributions among Vlax Roma groups, reflecting distinct demographic histories shaped by migration and endogamy [11]. Subsequent biomedical investigations demonstrate a substantial burden of health risk factors related to socioeconomic disadvantage and economic transition [20], including an increased prevalence of chronic non-communicable diseases and elevated mortality rates [20,21].

Further studies show that migration patterns and varying degrees of isolation among Croatian Roma groups are reflected in the distribution of genetic variants and in the carrier frequencies of rare disease-associated mutations [22]. Additional research highlights the high prevalence of osteoporosis in Croatian Roma from Baranja, emphasizing the need to investigate the association of low bone mineral density with specific lifestyle and reproductive factors in this population [23]. Across all age groups, low socioeconomic status and limited access to health care further contribute to unfavorable health outcomes [20].

More recent pharmacogenetic studies of *NAT* and *CYP2D6* genes confirm pronounced genetic differentiation among Croatian Roma groups arising from different degrees of isolation [17,24]. These findings illustrate how long-term demographic processes—including founder events, population bottlenecks, and genetic drift—have shaped group-specific genetic variation and led to the accumulation of rare variants in isolated Roma populations.

Within this broader framework of population structure and founder effects, population-specific analyses of clinically relevant genes are essential. However, despite extensive research on genetic and health characteristics of the Croatian Roma, data on polymorphisms in the *GJB2* gene, one of the most important genetic contributors to hereditary hearing impairment, have been missing.

Taken together, the complex migration history, socio-cultural isolation, and long-term endogamy of the Roma population also affect the distribution of other mutations responsible for autosomal disorders [25]. The uneven pattern of genetic variation has also been observed in the distribution of variants in the *GJB2* gene, the most significant gene in nonsyndromic hearing loss. In this study, polymorphisms of the *GJB2* gene were analyzed in three Croatian Roma groups—Vlax Roma from Medjimurje and Baranja, and Balkan Roma from Zagreb. Results of genotyping reveal three polymorphic SNPs, c.71G>A, c.457G>A, and c.380G>A, while c.109G>A and c.269T>C were monomorphic without altered alleles. Consistent with the existing knowledge of Roma genetic history [25], the results of this study confirm significant genetic heterogeneity among studied groups.

The c.71G>A, the most studied *GJB2* variant among Roma, known for its high frequency among Roma populations across Europe (e.g., Slovakia, Spain, Czech Republic), was also identified in this study, confirming the existence of a shared genetic heritage dating back to the proto-Roma period in South Asia [10]. The c.71G>A variant was originally identified in a Pakistani family [26] and subsequently reported across several Asian families [27,28,29], indicating an early emergence and wide dissemination of this *GJB2* allele. Available data indicate that this variant is the predominant DFNB1 allele in India, highlighting its major contribution to autosomal recessive nonsyndromic hearing loss in this population [30,31]. This observation is consistent with the high frequency of c.71G>A observed in Roma populations across Europe and reinforces the hypothesis that this variant was introduced into Europe during the historical migration of Roma from South Asia. Collectively, these findings highlight the importance of a population’s genetic history in shaping the variation spectrum of *GJB2* and underscore the contribution of c.71G>A to autosomal recessive nonsyndromic hearing loss in both South Asian and Roma populations. The different prevalence of this mutation in Roma groups throughout Europe is attributed to a strong founder effect, particularly pronounced in isolated populations with limited gene flow. Interestingly, this mutation was not recorded in the Croatian Roma group from Medjimurje, whose gene pool shows differences from other Croatia Roma group in various loci, e.g., [16,25]. The c.71G>A variant is categorized as pathogenic according to the ClinVar classification [32,33].

The c.380G>A variant is the most frequent among South Asians (gnomAD). Therefore, its presence in the Roma population probably predates their arrival to Europe. In the Croatian Roma population, its frequency ranges from 9 to 28% and shows significant difference among Roma groups. This variant affects the residue that is not highly conserved, suggesting its nonpathogenic nature [34,35]. Computational tools and conservation analyses of this variant do not strongly indicate its effect on the protein. In vitro studies suggest that the variant might influence the protein [36,37]; however, these studies may not accurately reflect the biological consequences of the variant. According to the ClinVar, the clinical significance of this variant remains uncertain.

c.457G>A is another *GJB2* variant present in highest frequency among South Asians among worldwide populations (gnomAD), suggesting its South Asian origin. Although present in more than 5% of contemporary South Asians, it is not detected in general European Roma populations, but only in studies involving patients with hearing impairment [13]. c.457G>A is present among the Croatian Roma population only in the Balkan Roma group with a frequency of less than 1%. Messe et al. [38] performed functional studies demonstrating that the mutants lost the ability to form functional gap junction channels; however, these findings have not yet been independently replicated. Mostly due to high prevalence in South Asian populations and a lack of functional evidence of pathogenicity, the ClinVar considers this variant benign.

Although the *GJB2* gene is the most common cause of recessive deafness in many populations, the presence of heterozygous cases without a second identified mutation, as well as findings of mutations in neighboring genes such as *GJB6*, indicate the need for broader genetic evaluation. Understanding the genetic basis of hearing loss in isolated and marginalized populations is essential not only for accurate diagnosis but also for the development of inclusive therapeutic approaches within precision medicine.

## 5. Conclusions

This study of the genetic basis of autosomal recessive nonsyndromic hearing loss in three Roma populations from Croatia reveals a high level of genetic differentiation between population groups, particularly with respect to the c.71G>A variant. These findings highlight the importance of characterizing population-specific mutational heterogeneity, which is essential for effective prevention, diagnosis and clinical management of autosomal recessive nonsyndromic hearing loss.

## Figures and Tables

**Table 1:** Genotype and allele frequencies in the total Croatian Roma population and in the three subpopulations separately (Balkan, Medjimurje and Baranja).

		** **	**Balkan**	**Medjimurje**	**Baranja**	**Total**	**X^2^**	*p*
			**N (%)**	**N (%)**	**N (%)**	**N (%)**		
p.Val153Ile c.457G>A rs111033186 13:20189125	genotype	G:G	175 (97.77)	127 (100)	131 (100)	433 (99.54)	2.9287	0.2312
		G:A	2 (1.12)	0	0	2 (0.43)		
		A:A	0	0	0			
	allele	A	2 (0.56)	0	0	2 (0.23)		
p.Arg127His c.380G>A rs111033196 13:20189202	genotype	C:C	146 (81.56)	66 (51.97)	88 (67.18)	300 (68.97)	34.6553	0.0547 *
		T:C	30 (16.76)	52 (40.94)	37 (28.24)	119 (27.36)		
		T:T	1	9 (7.09)	6 (4.58)	16 (3.68)		
	allele	T	32 (8.94)	70 (27.56)	49 (18.7)	151 (17.36)		
p.Trp24* c.71G>A rs104894396 13:20189511	genotype	G:G	163 (91.06)	127 (100)	125 (94)	415 (65.4)	10.5468	0.0051 **
		G:A	14 (7.82)	0	6 (4.58)	20 (4.6)		
		A:A	0	0	0			
	allele	A	14 (3.91)	0	6 (2.29)	20 (2.3)		

* Significant at *p* = 0.05; ** significant after Bonferroni correction *p* = 0.017.

## Data Availability

All data analyzed in this study are available upon request.
